# Biodegradable Scaffolds for Bone Regeneration Combined with Drug-Delivery Systems in Osteomyelitis Therapy

**DOI:** 10.3390/ph10040096

**Published:** 2017-12-12

**Authors:** Rossella Dorati, Antonella DeTrizio, Tiziana Modena, Bice Conti, Francesco Benazzo, Giulia Gastaldi, Ida Genta

**Affiliations:** 1 Department of Drug Sciences, University of Pavia, Viale Taramelli 12, 27100 Pavia, Italy; rossella.dorati@unipv.it (R.D.); Antonella.detrizio01@universitadipavia.it (A.D.); tiziiana.modena@unipv.it (T.M.); Ida.genta@unipv.it (I.G.); 2 Center of Health Technology, University of Pavia, Via Ferrata 1, 27100 Pavia, Italy; Francesco.benazzo@unipv.it; 3 Centre oh Health Technology (CHT), Via Ferrata 1, University of Pavia, 27100 Pavia, Italy; giulia.gastaldi@unipv.it; 4 Department of Molecular Medicine, University of Pavia, Viale Taramelli 2, 27100 Pavia, Italy

**Keywords:** bone regeneration, osteomyelitis, scaffold, gentamicin, antibiotics

## Abstract

A great deal of research is ongoing in the area of tissue engineering (TE) for bone regeneration. A possible improvement in restoring damaged tissues involves the loading of drugs such as proteins, genes, growth factors, antibiotics, and anti-inflammatory drugs into scaffolds for tissue regeneration. This mini-review is focused on the combination of the local delivery of antibiotic agents with bone regenerative therapy for the treatment of a severe bone infection such as osteomyelitis. The review includes a brief explanation of scaffolds for bone regeneration including scaffolds characteristics and types, a focus on severe bone infections (especially osteomyelitis and its treatment), and a literature review of local antibiotic delivery by the combination of scaffolds and drug-delivery systems. Some examples related to published studies on gentamicin sulfate-loaded drug-delivery systems combined with scaffolds are discussed, and future perspectives are highlighted.

## 1. Introduction

Tissue engineering (TE) aims to restore loss of tissue and organ functionality resulting from injury, aging, or disease [1]. Biomaterials, cells, and bioactive factors are commonly considered to be the key elements in the preparation of 3D tissue-engineered constructs for damaged tissue regeneration. Tissue regeneration is a process which takes place after an acute injury, and it can be achieved by the restoration or repair of tissue structure. Technically speaking, the word regeneration refers to the complete restitution of lost or damaged tissue, and repair involves restoring some original structure with scar formation. Regeneration is typical of tissues with high proliferation capacity such as the hematopoietic system, skin epithelia, gastrointestinal tract, and bone tissue. The process occurs because of the tissue’s ability to self-renew constantly after injury. Repair often consists of a combination of regeneration and scar formation by collagen deposition. The contribution of regeneration and scarring to the tissue repairs depends on the ability of the tissue to regenerate and on the extent of injury [2]. These processes (repair and regeneration) are regulated by several cell types, and consequently by different matrix proteins, growth factors, and cytokines.

TE has focused on designing constructs that support and promote the regeneration of several types of tissues, including skin, cartilage, bone, tendon, and cardiac tissues. The approach is to induce tissue regeneration at the defective site by providing a scaffold acting as artificial extracellular matrix (ECM). The scaffold assists cell attachment and subsequent proliferation and differentiation. If the artificial ECM is biocompatible, cells around the scaffold find a favorable environment for their infiltration into the scaffold and their proliferation. Scaffolds can be implanted in the body as acellular scaffolds, or in combination with cells and/or growth factors, cytokines, and genes (bioengineered scaffolds). The latter has the advantage of promoting faster tissue regeneration, especially when the tissue does not have inherent self-regenerating potential, as in pathological conditions. Growth factors (GFs) are important therapeutic agents for inducing regeneration in many tissues, such as skeletal muscle and neuronal, hepatic, and vascular tissues. Their direct injection into the damaged site is generally not effective because of their rapid diffusion from the injected site, as well as their enzymatic digestion and deactivation. These issues can be overcome by combining scaffolds with a drug delivery system (DDS). The DDS can promote a prolonged drug release both directly and selectively at the implantation site, and it can protect growth factors and protein molecules from degradation [3,4]. Beyond proteins, genes, and growth factors [5,6,7,8,9,10,11,12,13], drugs such as antibiotics and anti-inflammatory drugs are also of utmost importance for the success of tissue regeneration therapy. Antibiotic administration is fundamental in order to reduce infection risks during the implantation procedure and healing process or to treat pre-existing infections. Anti-inflammatory drugs reduce inflammation at the site of scaffold implantation [14,15,16,17], promoting the healing of damaged tissue.

The extensive literature available on this topic highlights how the most studied and suitable strategy involves the incorporation of drugs into scaffolds or their encapsulation in polymeric drug-delivery systems that can be combined with the scaffold. In these terms, biodegradable polymers are interesting and widely studied materials.

The present review is focused on the local delivery of antibiotic agents for treating severe bone infection, such as osteomyelitis.

Gentamicin is an aminoglycoside antibiotic which is extensively used for the treatment of many types of infections because it presents a wide bacterial spectrum. However, it has low bioavailability after oral administration and poor cellular penetration; in addition, internalized gentamicin molecules are accumulated in the lysosomal compartment, leading to the reduction of its activity. Following parenteral administration, gentamicin is excreted rapidly by glomerular filtration, resulting in a plasma half-life of 2 h in patients with normal renal function, and its half-life in the renal cortex is 100 h. Therefore, the administration of repetitive doses results in renal accumulation and nephrotoxicity. Moreover, the prolonged administration of gentamicin can cause ototoxicity due to free radicals formation. For these reasons, the local delivery of gentamicin is studied, and innovative preparations based on cement and polymeric beads are already on the market.

Local antibiotic delivery to bone can exploit scaffolds for bone regeneration as drug carriers. In this perspective, the infection treatment and tissue regenerative therapy can be combined in order to achieve reconstruction of the infected and/or necrotic bone tissue that has been surgically removed.

After the brief introduction, following chapters are dedicated to clarifying characteristics of scaffolds for bone regeneration and implantation, to explaining the cause and pathophysiology of osteomyelitis and its treatments, and to describing the advantages of combining an antibiotic (gentamicin) with polymeric scaffolds as filler of bone defects generated by bone resection following surgical treatment of osteomyelitis.

## 2. Scaffolds for Bone Tissue Regeneration

A scaffold for tissue regeneration is a structure which is able to support and/or promote tissue regeneration. It should possess a 3D and well-defined macro-architecture and micro-architecture with an interconnected pore network. It should be biocompatible, and its mechanical properties should be similar to those of original bone tissue. Scaffolds for bone regeneration can be made of diverse materials: polymers or polymers combined with calcium phosphate minerals as hydroxyapatite, or to other compounds such as single-walled or multi-walled-carbon nanotubes. They should possess all requirements of injectable products, such as sterility and apyrogenicity [18,19,20,21], because they are intended for implantation into the human body. Biocompatibility is an unavoidable requirement of a scaffold: if it is a temporary scaffold, it should be biocompatible and bio-resorbable with controllable degradation and resorption rate. They can also provide controlled release of specific bioactive factors in order to enhance or guide the regeneration process [22,23].

Bone tissue presents anisotropic behaviour because its strength depends on orientation of the applied load and resistance to high pressure/loadings, and the resistance depends on the positioning of bone in the human body and its size. For these reasons, specific scaffold structure, shape, and composition may be useful, according to the bone restoring needs. All these variables (shape, structure, and composition) should be balanced in order to find the combination that perfectly matches with the properties and functions of the damaged bone. This means a significant number of combinations among polymers, minerals, and other materials need to be evaluated.

Non-engineered (acellular) scaffolds available to treat non-healing bone fractures are defined as autografts, allografts, and metallic prosthetic implants; autografts and allografts are implanted by invasive procedures, which do not always end with the healing of damaged tissue. Critical issues related to allograft and autograft implants are identified as high risk of infections, the painful process needed to harvest bone graft from the iliac crests, and long post-operative recovery. In addition, autograft and allograft implants are made of avascular and non-viable tissue; they do not carry cellular components of bones, resulting in a lack of bone remodelling. The percentage of failure of these procedures is up to 25–35% when the immune system induces graft rejection [24,25].

Even though metals are not biodegradable materials and do not promote bone tissue regeneration, they are widely used in implants for bone healing, and are worth mentioning. The main advantages of metallic implants are stiffness and high load-bearing mechanical properties combined with an absence of body immune response [26]. For these reasons, they are used frequently in bone surgery addressed to tissue restoration—above all in the cases of long bones that must withstand high compressive and elastic forces. The most-used metals are titanium and its alloys, and stainless steel; they seem to be useful, but require invasive procedures for implantation. Classic metal implants do not promote osteoinduction or osteoconduction, and they do not improve bone regrowth. In many cases, the metal implant is withdrawn when bone healing is completed, implicating a second surgery that is associated with pain, high risk of infection, and further days of immobilization. Problems associated with stress shielding, fatigue, and loosening of implant are often highlighted with metal implants, leading to a second substitution surgery.

Recent experimental works have addressed the modification of the structure and composition of titanium implants in order to obtain implants with osteogenic properties. Benazzo and coworkers highlighted that trabecular titanium can be useful to induce in vitro osteogenic differentiation of adipose-derived stem cells without osteogenic factors [27,28], while Böhrnsen and coworkers successfully evaluated the hypothesis of binding bone morphogenetic protein 2 recombinant (rhBMP-2) to the surface of titanium implants. They demonstrated that the rhBMP surface-modified titanium implants could significantly enhance in vivo osteogenic differentiation in peri-implant bone, especially in the early phase of osseointegration [29]. Carmargo and coworkers demonstrated that novel alkali-based surface modification enhances in vitro mineralization as well as in vivo bone formation using a titanium implant [30].

Stress shielding involves loss of bone integrity due to the high stiffness of metal bone implants. The modification of implant topography represents one of the most promising approaches for reducing bone integrity loss caused by stress shielding. Strategies to reduce this drawback are reported in the literature [31], including the modification of implant topography [32].

As long as the focus of the present paper is concerned, the antibacterial activity of metal implants can be achieved by surface coating with ions, as demonstrated by Shivaram. The principal issue highlighted with this technique is to get a stable coating assuring a long-term release of ions eliciting the antibacterial effect [33]. Chen and coworkers obtained suitable antibacterial activity on streptococcus mutants by coating the titanium surface with metal–organic framework films based on Zn^2+^. The films composed of zeolitic imidazolate framework-8 (ZIF-8) crystals with nanoscale and microscale sizes (nanoZIF-8 and microZIF-8) were prepared on porous titanium surfaces by hydro- and solvo-thermal methods, respectively [34].

Biomaterials—namely biocompatible polymers ceramic and bioglass—have the advantages that they integrate in the surrounding tissue without being rejected and minimize host reactions at the implant site. This is an important property underlined by several authors, including F.D. Williams: “*The biocompatibility of a scaffold or a matrix for a tissue engineering product refers to the ability to perform as a substrate that will support the appropriate cellular activity including the facilitation of molecular and mechanical signaling systems, in order to optimize tissue regeneration, without eliciting any undesirable local or systemic responses in the eventual host”* [35]. Materials with these characteristics seem to accelerate tissue healing, and moreover an explant surgical procedure is not required when polymeric scaffolds are used, as the biomaterial is re-absorbed or completely integrated with new tissue.

Depending on its composition, polymer-based scaffolds can be classified as natural scaffolds, synthetic scaffolds, unblended scaffolds, and composite scaffolds. The desired longevity of the polymeric scaffold implicates the use of bio-inert or biodegradable polymers, and their stability involves the application of unblended or composite polymers, whereas the desired cellular interactions guides the choice of naturally- or synthetically-derived polymers [36,37].

### 2.1. Natural Polymers

Natural polymers have good biocompatibility, and they can be easily modified and processed into various structures [38]. However, their provenance from animal sources can increase the risk of pathogen transmission and immune-rejection; moreover, their poor mechanical strength does not assure whole protection of seeded cells, slowing the healing process and in the worst-case leading to implantation failure. An example is represented by collagen, which is used unblended for cartilage regeneration and in association with other polymers or materials (composite scaffolds) for bone tissue regeneration. Collagen, hyaluronic acid (HA), carboxymethyl cellulose (CMC), and chitosan are some of the most studied natural polymers for bone regeneration [37,38].

Collagen is a fibrous protein which is widespread in the animal and human body. Collagen properties depend on in its fibrillar (e.g., type I, II, III, V, XI collagen) or non-fibrillar structure (e.g., type IV, VIII, IX, X, XII, XIV, XIX, XXI collagen); it is the main ECM component in mammalian tissue. As far as bone regeneration is concerned, collagen crosslinking degree and crosslinking agents are studied to improve its mechanical properties [39,40].

Hyaluronic acid (HA, hyaluronan) is a natural, hydrophilic, non-immunogenic, biodegradable, non-sulphated glycosaminoglycan. HA is highly concentrated in early bone fractures, and it effectively supports bone growth when is blended with other osteoconductive molecules; however, the reduced viscoelastic mechanical features make HA unsuitable for the regeneration of trabecular bone.

Carboxymethyl cellulose (CMC) finds application in cellulose-based natural scaffolds for tissue regeneration; its structure is similar to that of chitosan. Sodium CMC is commonly used because it is a water-soluble polymer. The polymer is hydrophilic and viscoelastic, and these properties make CMC an eligible material for composite scaffolds, which need to overcome the drawbacks of osteo-inductivity and -conductivity [41].

Chitosan is a polysaccharide made of d-glucosamine and *N*-acetyl-d-glucosamine linked by β(1,4) glycosidic bonds α(1–4)-2-amino-2-deoxyβ-d-glucan. It has been widely studied for applications in tissue regeneration as a result of its promising properties [42,43,44,45]. The polymer is a deacetylated form of chitin which is mostly obtained from the deacetylation of chitin extracted from crustaceous shells, but chitosan produced by mushrooms is also available on the market. Chitosan deacetylation degree is fundamental for its reactivity and properties. Chitosan is soluble in water at acidic pHs, and is insoluble at neutral pH.

The hydrophilicity and the positive charge of chitosan are important properties allowing the polymer to interact with negatively-charged polymers, with negatively-charged macromolecules, and with certain polyanions in the aqueous environment. These interactive forces and the resulting sol–gel transition stages have been exploited for nano-encapsulation purposes. Moreover, the positive charge of chitosan improves its adhesion to the human and animal mucosal surfaces; this property has drawn attention to chitosan for use in mucosal drug delivery.

The potential of chitosan for this specific application has been further enforced by the demonstrated capacity of chitosan to open the tight junctions between epithelial cells of well-organized epithelia. Together with mucoadhesion, this last property is responsible for the improvement of transmucosal permeability and enhanced transport through the paracellular pathway shown by chitosan nanoparticulate drug delivery systems. The interesting biopharmaceutical characteristics of this polymer are accompanied by its well-documented biocompatibility and low toxicity. Chitosan’s drawbacks are: (i) pH-dependent solubility—the polymer is soluble only at acidic pH below five and at basic pHs, while it is insoluble at neutral pH. This drawback is overcome through chitosan salts such as chitosan hydrochloride or chitosan glutamate that show pH-independent solubility; (ii) slight polymer toxicity due to its positive charge given by NH_2_ groups.

Chitosan has the ability to form thermosensitive hydrogels by interaction with polyanions. Glycerophosphate and tripolyphosphate are the most investigated polyanions for preparing hydrogel as a drug delivery system or as a scaffold for soft tissue regeneration. Injectable in situ forming gels have been proposed for cartilage repair and as filling material for bone repair [36,46,47,48]. Case study involving this topic will be further developed in Section 5. Chitosan undergoes enzymatic degradation in vivo, and its degradation products enter the human metabolic cycle. The in vivo performances of chitosan can vary depending on its molecular weight, deacetylation degree, and functionalization with chemical groups (e.g., trimethylated chitosan).

### 2.2. Synthetic Biodegradable Polymers

The advantage of this type of polymer is their versatile behaviour. Their properties such as mechanical strength and biodegradation rate depend on their molecular weight and composition, which can be tailored according to specific necessities. However, a lack of biological signals and the resulting lack of cell response are frequent critical issues of this type of polymers.

Synthetic polymer degradation is mainly driven by hydrolysis, while natural polymers are degraded mostly through enzymatic pathways or combined with hydrolysis. The most-studied and used synthetic polymers are poly-alpha-hydroxy acids and derivatives, polycaprolactone (PCL).

Poly-alpha-hydroxy acids such as polylactide (PLA) and related copolymers polylactide-*co*-glycolide (PLGA) have been largely studied for their suitable properties such as biocompatibility, safety (in terms of possibility of disease transmission), and absence immunological reactions. However, their poor mechanical properties limit their use in fractures involving high load-bearing bones. They degrade through hydrolysis, releasing lactic and glycolic acid oligomers and monomers that are eliminated through the metabolic pathways. PLAs are hydrophobic, while PLGA copolymers are more hydrophilic thanks to the presence of glycolic acid. Hydrophilicity accelerates polymer degradation since it accelerates polymer and scaffold wettability. PLGA 75/25 are more cell-inductive and conductive than PLGA 50/50 or 85/15. Due to the biodegradable nature of these polymers, the scaffolds made of PLGA are defined as temporary structures (dynamic scaffold). The polymer degradation rate of a temporary scaffold should be synchronized with the tissue growth process. This is an important point in order to assure suitable support of tissue growth, disappearing whenever the tissue has formed [49,50]. The Food and Drug Administration (FDA) and European Medicines Agency (EMA) approved both PLAs and PLGAs polymers for implantation in the human body. This currently represents an incomparable advantage for manufacturing and marketing authorization of medical devices such as implantable scaffolds.

Polycaprolactone (PCL) is a synthetic polymer which has been well studied for bone and cartilage repair as a consequence of its stability and very long degradation times. Due to its low glass transition temperature (Tg), the polymer is in the rubbery state at 37 °C, showing optimal plasticizing features. PCL is often combined with tougher polymers because it is used to make scaffolds for bone regeneration, and it can be copolymerized or blended with PLA that presents a crystalline structure. The FDA approved PCL for implantation in the human body as a drug delivery device and suture material.

Other synthetic biodegradable polymers studied for application in bone regeneration are poly propylene fumarate (PPF), polyanhydrides, and polyphosphazenes.

PPF is a biocompatible, biodegradable, and osteoconductive biomaterial. Its properties depend on its molecular weight and molecular structure, as well as on crosslinking degree. This polymer is a suitable component of both preformed solid scaffolds and injectable scaffolds. Its degradation by hydrolysis leads to fumaric acid, which is easily excreted by the body. Polymer degradation can be manipulated through crosslinking degree and type of crosslinking agent.

Polyanhydrides—namely aliphatic and homo-polyanhydrides—rapidly biodegrade through hydrolysis, and for this reason they are not suitable for applications in tissue engineering. Photocrosslinkable polyanhydrides could be used for orthopedic applications as injectable polymers to be crosslinked in situ; nevertheless, their use is limited by their hydrolytic instability. Polyanhydrides are used for controlled drug delivery; they have been copolymerized in order to increase their hydrophobicity and decrease their biodegradation rate. Poly (1,6-bis-(*p*-carboxyphenoxy hexane)-*co*-(sebacic anhydride) (PANH) is a polyanhydride copolymer proposed by Ku and co-workers. They prepared composite matrices in which PANH polymer was embedded into poly(ɛ-caprolactone) grafted to hydroxyapatite (PCL-gHAP). The PCL-gHAP/PANH composites demonstrated stability for at least 4 weeks with suitable mechanical properties, and in vivo studies highlighted improved functionalities of HAP in terms of new bone formation [51].

Polyphosphazenes are biocompatible high molecular weight polymers; their advantage is their degradation rate, which can be controlled by the substitution percentage and side chain nature. Laurencin and co-workers investigated these polymers for bone regeneration applications. They demonstrated that poly(glycine ethyl glycinato)_1_(phenilphenoxy)_1_ phosphazene combined with PLGA neutralizes acidic polyesters degradation products, decreasing PLA degradation rate. Moreover, the glycilglycine derivatized polyphosphazene was demonstrated to be highly biocompatible and osteocompatible [52,53].

### 2.3. Ceramics and Bioglasses

Ceramics are neither metallic nor organic compounds; they show good strength and resistance to deformation, as well as suitable osteoconductive properties. They are fragile by nature and they are combined with polymers to overcome the fragility issue, keeping all their advantages. Hydroxyapatite (HAP), β-tricalcium phosphate (β-TCP) and biphasic calcium phosphate (BCP) are the most common ceramic types. They are capable of integrating into bone structures being resorbed, and support bone in-growth without dissolving [54,55].

Bioglasses are another important class of materials in bone regeneration. The compounds have inorganic composition, and the original one was based on Na_2_O-CaO-SiO_2_-P_2_O_5_.

Today, phosphate-based, silicate-based, and borate-based bioglasses are on the market (e.g., Novabone^®^). Extensive research has been conducted on these materials, starting from their first development and studies by Hench in 1969 [55,56,57]. Their trade name Bioglass^®^ highlights the bioactive behaviour and main advantage. In fact, the material was demonstrated to bind bone tissue by a mechanism attributed to formation of a hydrocarbonate apatite (HCA) layer on the glass surface. HCA is similar to hydroxyapatatite bone component and promotes interaction with the collagen fibrils of damaged bone. The resulting integration with the host bone includes cell differentiation and excretion of bone extracellular matrix with bone mineralization. Moreover, bioglass dissolution product induces osteogenesis [58,59,60]. An important issue is the bioglasses’ degradation time and the fate of degradation products, which are glass particles. Degradation rate depends on bioglass scaffold morphology, structure, and implantation site, but is usually quite slow (12 months or longer) with the release of glass particles of different sizes. Authors differentiate between the fate of small particles with diameter <300 μm, which dissolve and are taken up by osteoclasts, and that of larger particles >300 μm, which remain longer in the body. Glass degradation releases silicon (Si) that can increase Si levels in the body. Although silicium is an inert compound, its serum level should be controlled and should not overcome its saturation level. The main drawbacks of bioglasses seem to be their slow degradation rate, increase of Na^+^ and Ca^2+^ concentration detected at the site of bioglass implantation, and difficulty in processing the material in the form of a 3D scaffold due to the scarce sintering ability of the glass [61]. In a recent paper, Mancuso and co-workers addressed the issues and developed novel silicate phosphate and borate glasses containing various oxides in diverse molar percentages, such as MgO, MnO_3_, Al_2_O_3_, CaF_2_, Fe_2_O_3_, ZnO, CuO, Cr_2_O_3_. The authors thoroughly characterized the materials for their physical, chemical, and biological properties, with promising results addressed to tissue regeneration [62].

Historically, the studies of these materials restrained the concept of biomaterial to a material whose interaction with the human body is capable of eliciting a specific biological response [63]. In the paper of Jones, the author nicely describes Bioglass^®^ history from its discovery, Bioglass^®^ advantages, the material’s osteoconduction and osteoinduction properties, and introduces new bioglass composite hybrid materials. Composites with increased stiffness and mechanical properties are obtained by blending bioglass particles with biodegradable polymers such as the well-known PLA, polyglycolic acid (PGA), PLGA, and PCL, whose characteristics are reported in Section 2.2 [64,65,66,67,68,69].

More recently, the antibacterial effect of purpose-modified bioglasses has been studied. According to the authors, the antibacterial effect of bioglass depends on the bioglass structure and scaffold morphology. Indeed, it can be improved by doping the bioactive glass with zinc and strontium or silver ions [59,60,61].

Rivadeneira and co-workers evaluated the influence of bioglass on vancomycin release from gelatin films. They showed that bioglass addition to gelatin films modulates the films’ degradation and the antibiotic release rate, without interfering with antibacterial effect of the antibiotic [70].

## 3. Scaffolds and Drug Delivery

Polymer matrix or scaffold are 3D platforms that can serve the dual purpose of cell support and cells/growth factors (GFs)/drugs delivery. Porosity is perhaps the most important structural scaffold requirement, and includes macropores (>50 nm, <300 nm) useful to cell penetration and tissue ingrowth, and smaller pores such as micropores (<2 nm) and mesopores (>2 nm, <50 nm) which allow nutrient transport and waste of metabolic products, permitting cell growth. In the case of drug-loaded scaffold porosity, micro- and meso-pores contribute to drug release by diffusion. Whenever scaffolds are made of biodegradable polymers, scaffold biodegradation also contributes to the release of a loaded drug. Scaffold degradation rate is a very important parameter to be set, in order to achieve suitable control of drug release. Moreover, scaffold biodegradation should be synchronized with the rate of tissue growth.

**Figure 1:**
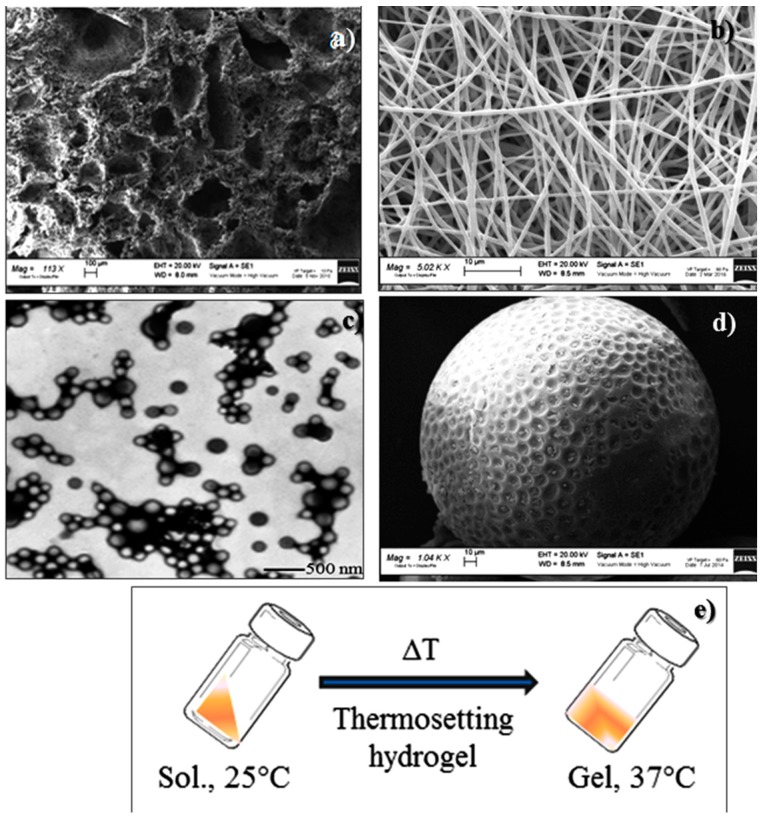
Examples of polymeric scaffolds for tissue regeneration/drug delivery: (**a**) 3D-porous matrix; (**b**) nanofibrous matrix; (**c**) nanoparticles; (**d**) microspheres; (**e**) thermosensitive hydrogel.

Functionality enhancement of these already complex matrices has been achieved by incorporating drugs or drugs encapsulated into drug delivery systems. The drug-releasing scaffolds permit local delivery of an adequate dose of bioactive molecules for a desired period, minimizing active agent release to non-targeted sites, supporting and promoting tissue regeneration, which normally occurs over a long time span. From this perspective, TE can be viewed as a special case of controlled drug delivery combined with scaffolding materials. Drug-releasing scaffolds are new multifunctional platforms able to achieve drug delivery to specific sites with high loading and efficiency, and control the tissue regeneration process. Different forms of polymeric scaffolds for drug delivery are available, such as: (a) 3D porous matrices; (b) electrospun nanofibrous matrices; (c) nanoparticles; (d) porous microspheres; (e) thermosensitive hydrogels (Figure 1).

Cells can be seeded onto the 3D polymer scaffolds or 3D-porous matrix in order to achieve an engineered tissue. Moreover, cell delivery can also be achieved through their microencapsulation, as commonly performed into alginate microcapsules [71,72,73].

Cells can act as a hormone delivery system; for example, the encapsulation of islets of Langerhans to produce insulin [74,75,76,77]. Indeed, drugs (growth factors and genes, antibiotics, anti-inflammatory drugs) can be integrated within scaffolds by simply interspersing them into the polymer matrix. Examples in the literature report biodegradable hydrogels acting simultaneously as scaffolds and controlled delivery systems. In addition, the hydrogel system can release more than one type of growth factor at the same time or in a time-ordered way.

**Figure 2:**
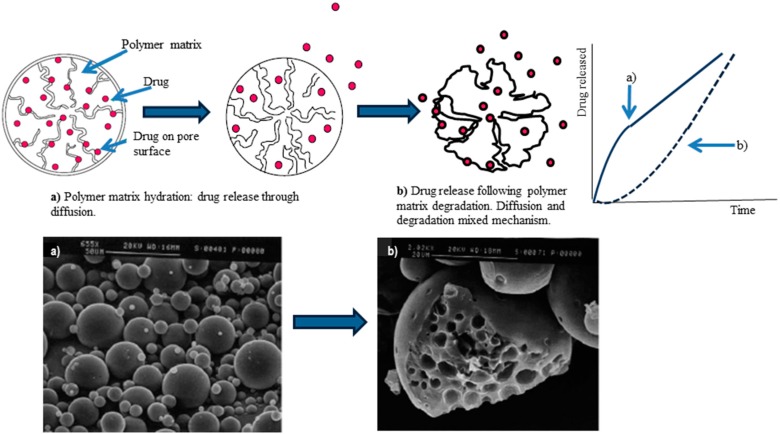
Scheme of “in vitro” drug release mechanisms through biodegradable polymers microspheres and correlation with scanning electron microscopy images of the drug-loaded microspheres: (**a**) immediately after incubation; (**b**) 20 days after incubation.

Depending on the incorporation method used and the biomaterial characteristics (as discussed above), drug release rate may be controlled by various processes, such as diffusion, polymer erosion or degradation, and swelling of polymer followed by diffusion. The different processes can participate at the same time, or one process can prevail. When drugs are immobilized by covalent immobilization (e.g., proteins), the release mechanism involves chemical/enzymatic scissions. Drugs’ release profile can be altered by modifying polymer properties or adjusting scaffold physical and chemical properties such as porosity, pore size, and shape (e.g., tortuosity), polymer crosslinking degree, and degradation rate. Additionally, drugs and cells can be encapsulated into biodegradable particulate systems having the potential to be retained in specific tissues, providing sustained release [5,6,7,8,9,10,11,12,13,14,15,16,17,78]. Figure 2 shows a scheme of drug release mechanism from polymeric biodegradable microspheres that combines drug diffusion through a polymer matrix and polymer degradation. The two mechanisms can be tailored depending on the desired drug release rate, exploiting formulation characteristics. The selection of polymer or polymer blends are of the utmost importance to achieve the suitable drug release rate.

Biomaterials for drug delivery can be designed in various morphologies (e.g., micelles, vesicles, particles, tubes, scaffolds, or gels) and architecture (reservoirs or matrices). Drugs can be safely encapsulated in non-cytotoxic and biodegradable synthetic polymers such as polylactic acid (PLA), polyglycolic acid (PGA), their copolymer polylactic-*co*-glycolic acid (PLGA), poly-ε-caprolactone (PCL), polyethylene, polymethylmethacrylate (PMMA), or natural hydrogels such as alginate, gelatin, fibrin, collagen, and chitosan. When natural hydrogels are used, chemical or physical cross-linking give the ability to control drug diffusion. Moreover, drug-loaded micro/nanoparticulate systems can be further incorporated into a hydrogel or porous scaffold in order to simultaneously or sequentially deliver one or more drugs with diverse activities (e.g., growth factors and anti-inflammatory drugs). Composite systems of diverse materials combine the individual benefits from each material to efficiently deliver drugs, and may offer the most beneficial clinical outcomes. Moreover, nanotechnology development has focused on nanoparticle delivery systems with diameters between 50 to 700 nm as carriers for therapeutic agents, able to penetrate through capillaries into cells and enabling new tissue regeneration [79,80,81,82,83,84].

Interestingly, many biomaterials used for drug delivery systems are the same as for scaffold manufacturing (see Section 2), creating a perfect interaction between drug delivery and tissue regeneration research areas.

## 4. Osteomyelitis and Osteomyelitis Therapy

Osteomyelitis is an infection typical of bone and is caused by pyogenic bacteria including certain strains of mycobacteria and fungi. Typical microorganisms which are prevalent causative agents of the disease are *Staphylococcus aureus*, followed by *Pseudomonas* and *Enterobacteriaceae, Salmonella, Clostridium,* and *Pasteurella multocida*; osteomyelitis involving diabetic foot is caused by polymicrobial infections; osteomyelitis following implantation of prosthesis is prevalently caused by *Staphylococcus aureus* [85]. Based on pathogenic mechanism and infection source, osteomyelitis can be divided into three categories: (a) microbial infection due to trauma, surgical operation, or incorporation of prosthetic joint; (b) infection with hematogenous spread, most frequent in vertebral bone and children; (c) infection associated with vascular insufficiency as it happens in diabetes mellitus and/or peripheral vascular disease. Osteomyelitis progression involves inflammation, subsequent bone loss, and spreading of bacterial infection to soft tissue. Three infection sub-categories can be recognized, such as acute, which develops within 10 days; sub-acute, which develops within two weeks to one month; and chronic, which keeps after several months. Acute osteomyelitis can occur from bacterial presence in the blood with edema, local anoxia, and pus formation. Chronic osteomyelitis exhibits osteonecrosis, the formation of a large area of devascularized sequestrum, and generally is due to untreated or incorrect treatment of acute phase. Although infection of bones is found to be quite rare in average conditions, foreign materials implantation and bone damage like trauma or open fracture can often lead to osteomyelitis. The problem is significant in the case of periprosthetic implant infections, known as septic failures, because they lead to prolonged hospitalization and have high risk of relapses with poor healing outcomes. Moreover, septic failures are associated with high costs involved in the treatments, which have been reported to amount to $70,000/per episode in the USA [86,87,88,89]. A high risk of osteomyelitis infection is observed in patients suffering from diabetes, hepato-renal failure, and immune suppression.

Moreover, it has been reported that infection rate is lowest after primary joint replacement (1%) with respect to infection risk associated with open fractures (23%). In acute infection, bacteria after entering and being phagocytosed release toxic oxygen radicals and proteolytic enzymes which cause lysis of surrounding tissue. Inflammation of surrounding blood vessels leads to increased intraosseous pressure, reducing blood flow in the infected area. Eventually, a non-vascularized sequestrum is generated from necrotic bone tissue formation, and it can create a fistula that breaks through skin.

Conventional therapy of osteomyelitis includes surgical debridement, removal of implant and necrotic tissue, blood supply and soft tissue restoration, and oral/systemic antibiotic administration. Surgical removal of sequestrum and infected bone tissue involves additional surgery that can lead to more chances of infections with elevated medical costs and subsequent bone loss with difficult healing. As an alternative, antibiotic therapy is the traditional therapy for chronic osteomyelitis treatment. Systemic antibiotic therapy needs high doses to successfully eradicate bacteria, with subsequent increase of systemic toxicity and expensive hospitalization costs. Intravenous administration of glycopeptide drugs like vancomycin, daptinomycin, and teicoplanin demonstrated limited penetration into bone tissue [90]. Although several antibiotics exist, a good solution to treat this infection has not yet been found. The poor vascularization of bone tissue hinders antibiotics penetration, facilitating increased bacterial resistance. Moreover, traditional antibiotic therapy is sometimes unable to control the infection due to bacterial defence mechanisms, such as bacteria survival in a dormant state inside osteoblasts, and/or developing biofilm. Moreover, bacteria in a planktonic phase can become a sessile form with a high metabolic rate and rapid multiplication, reducing their sensitivity to antibiotics by a factor of 10^3^ [91].

Oral therapy of osteomyelitis is performed with fluoroquinone drugs. Ciprofloxacin, moxifloxacin, levofloxacin, clindamycin, metronidazole, phosphomycin, and trimethoprim-sulphamethoxazole are the most common antibiotics used for this purpose. Therapy should be continued for 12–16 weeks at very high antibiotic doses [92].

Local delivery of antibiotics has emerged as a new therapeutic strategy to overcome the limitations and effectively manage osteomyelitis [91,93,94,95,96]. Local application justifies elevated antibiotics concentration in an avascular zone able to destroy the persistent infection originated from microorganism residuals in biofilms [97]. Local antibiotic delivery has many advantages over parenteral and oral administration in osteomyelitis therapy: the antibiotic reaches much higher concentration at the site of action, while keeping systemic antibiotic concentration at a minimum level, and adverse side effects commonly occurring with high systemic doses of antibiotics can be avoided. This improvement in the healing process leads to an important decrease of adverse hypersensitivity reactions, a shorter hospital stay with reduced costs and improved patient compliance. Local antibiotic delivery systems can be non-biodegradable or biodegradable. The gold standard for treatment of infection associated with bone and soft tissue are antibiotic-loaded carriers based on inert materials such as cements or beads of polymethylmethacrylate (PMMA) impregnated with antibiotics able to release sustained high levels of antibiotic at a local administration site [98,99,100]. PMMA bead chains impregnated with antibiotic are frequently used for arthroplasties and muscoskeletal infections. They are available on the market with different antibiotic dosages and are uniform in size (about 7 mm in diameter). However, poor antibiotic elution and drug instability at high temperature when PMMA beads are obtained through polymerization represent some issues. Because of their stability at high temperature, aminoglycosides including streptomycin, gentamicin, amikacin, and tobramycin are the most used antibiotics to impregnate PMMA beads. However, antibiotic delivery by inert carriers such as PMMA beads results to be incomplete, causing a persistent release of low amounts of antibiotics that enhances the risk of antibiotic resistance. Moreover, PMMA can trigger immune response and thus some cytotoxic effects, and a second surgical intervention is needed to remove the exhausted PMMA beads.

Several authors investigated elution kinetics and antimicrobial effects of diverse antibiotics loaded into cements, bioglasses, and/or polymer systems [83,100,101,102,103,104,105,106,107,108,109,110,111,112,113,114,115,116,117,118,119]. Vancomycin, gentamicin, rifampicin, and moxifloxamicin showed excellent results with initial high elution rates followed by sustained elution rates for the entire experimental time, as well as keeping antimicrobial effect in situ. On the other hand, elution rate of etrapemen, meropenem, and daptinomycin decreased after 4 days. Antibiotic type and bioavailability influence drug elution, also depending on structural characteristics of bone grafts and cement [101,102,103,104,105,106,107,108,109,110,111,112,113]. Indeed, bioglasses—alone or in combination with biodegradable materials or metals—are proposed as antibiotic carriers for local therapy in osteomyelitis [104,105,106].

## 5. Biodegradable Scaffold-Drug Delivery Systems for Osteomyelitis Treatment

As an alternative, biodegradable materials are widely studied by researchers. Biodegradation time must be correlated to infection status and amount of tissue to be reconstructed or repaired. Antibiotic delivery can be more effective using biodegradable systems if compared to antibiotic delivery by inert carriers such as PMMA beads, because complete and effective drug release can be achieved. Moreover, no additional surgery is required to extract the exhausted carrier from the body, representing an important advantage for both patient and physician. Biodegradable devices for antibiotics delivery can be divided into three categories:

1. Proteins such as collagen, gelatin, and thrombin. Collagen-based materials are usually prepared from skin or tendon of animals and can provide sufficient stimulation for bone regeneration with osteoblast proliferation and increased mineralization. Collagen is an elemental part of connective tissue, it is found in all organs, and it does not induce toxicity. Moreover, drug content and elution properties depend on system porosity. In recent studies, local administration of antibiotic-loaded collagen sponges, associated with parenteral therapy, showed promising results for open fracture treatment [106].
2. Synthetic polymers like PLA, PLGA, PCL, their copolymers and pegylated derivatives. They are highly compatible with several antibiotics such as ampicillin, gentamicin, and polymixin-B. These materials can degrade very slowly (months or even years) at physiological pH, providing sustained release of antibiotics. Moreover, drug elution kinetics from these synthetic polymers can be modulated by changing physical, biochemical, and molecular structural properties of the polymer [108].
3. Bone graft materials and substitutes. Some antibiotic-loaded bone grafts are available on the market, namely Simplex^®^P, Osteoset-T^®^, Collatamp^®^, Septocoll^®^, Septopal^®^, Herafill^®^ beads, and Stimulan^®^ [109]. Antibiotic can be added by directly mixing antibiotic powder to bone graft or soaking bone graft in an antibiotic solution. Due to its low immune reaction, structural properties, and easy reabsorption, calcium sulphate is also used to manage chronic osteomyelitis, and Osteoset-T^®^ is a product on the market. Local application of calcium sulphate impregnated with tobramycin and vancomycin is effective to reduce infections due to implantation of prosthesis, also preventing colonization of bacteria and subsequent biofilm formation.

In summary, literature on the topic shows that correct use of antibiotics is very important to reduce morbidity and mortality from osteomyelitis; a gold standard is not currently available on the market; biodegradable composite systems combining an antibiotic delivery system with a scaffold able to improve tissue regeneration are envisaged. To the best of our knowledge, limited and controversial data are available on the performance of these new composite systems in animals and humans suffering from osteomyelitis, and still more work and study is needed to improve solutions for treating osteomyelitis [107,110,111,112].

The following section is dedicated to an example of research on the topic. Dorati and co-workers [116,117,118] carried out some research focused on the development of new biodegradable drug delivery systems combined to a biodegradable scaffold in order to locally administer gentamicin and promote prolonged release at infected tissue. Gentamicin is known to be a suitable antibiotic for osteomyelitis therapy; nevertheless, the high nephrotoxicity of gentamicin limits its use by systemic administration. This represents the rationale for formulating gentamicin in scaffolds for local administration.

**Figure 3:**
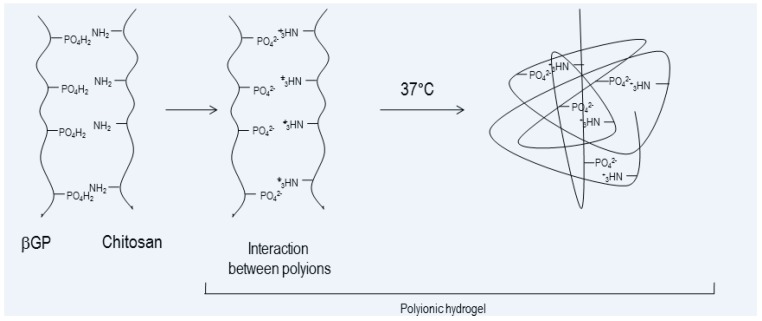
Schematic representation of gelification process between chitosan and β-glycerophosphate (β-GP).

The research involved a formulation study on injectable thermosetting composite scaffolds (ITCSs). ITCSs were newly prepared by combining a biodegradable and biocompatible thermogelling polymeric solution based on chitosan and supplemented with bovine bone substitute granules (BBS, natural hydroxyapatite) of 1–2 mm size (Geislich Orthoss^®^ granules). Chitosan (CHS) plays an important role in regenerative medicine; its adhesive nature and bacteriostatic properties are very important in osteomyelitis treatment [116]. The ITCS exploits chitosan’s ability to form a thermosensitive gel at 37 °C in the presence of β-glycerophosphate (β-GP, Figure 3). β-GP is a catalyst to induce sol–gel transition of chitosan solution at physiological environment at 37 °C, and it promotes bone regeneration. The presence of BBS organic component affects the physico-chemical features of ITCS, such as porosity, interconnectivity, mechanical properties, and biofunctionality. ITCS was lyophilized, obtaining a solid moldable 3D scaffold. The composite 3D matrix resulted in high water retention ability and hydrophilic nature, promoting cell attachment and infiltration. The polymeric component of ITCS was susceptible to degradation during the in vitro incubation in simulated physiological conditions (DMEM, pH 7.4, 37 °C) without showing remarkable toxicity caused by degradation products. Moreover, ITCS showed excellent in vitro physical stability, even after mechanical stress. The features made ITCS the starting point for an antibiotic drug delivery system combined with scaffold [22]. Literature reports gentamicin-loaded drug delivery systems based on borate bioactive glass, bone cement and polymeric devices made of hyaluronic acid, poly (l-lactic-acid) or poly (d,l-lactide–*co*-glycolide) [104,107,112,113,114,115], but chitosan combined to natural bovine bone substitute has not been deeply investigated yet, to the author’s knowledge.

The research work was a thorough investigation of biodegradable thermosetting hydrogel containing gentamicin sulphate for improving chronic osteomyelitis treatment. Gentamicin sulphate loading was evaluated either by directly loading the antibiotic into the ITCS and 3D moldable composite scaffold (mCS), or by manufacturing gentamicin-loaded microspheres and embedding them into the mCS: the injectable formulation and the moldable scaffold loaded with gentamicin sulphate or gentamicin sulphate microspheres were studied [116,117,118]. Gel forming was affected by gentamicin according to co-solute effect on sol/gel transition, limiting the loading amount of antibiotic.

Gentamicin sulphate addition to mCS in the selected quantities did not interfere with scaffold water uptake and retention capability. Gentamicin in vitro release was completed in 4 h, useful for exerting immediate antibacterial effect, thus limiting risks of infection during open surgeries. Good results in terms of cell seeding and proliferation were obtained. Antimicrobial effect, tested on *E. coli ATCC 10356* showed 24 h bactericidal effect, with superimposed bacteriostatic effect of chitosan in the first 4 h [118].

Gentamicin-loaded polylactide-*co*-glycolide-co-polyethyleneglycol (PLGA-PEG) microsphere were prepared and investigated by the authors. PLGA-PEG was selected in order to improve gentamicin sulphate content in the microspheres, thanks to higher polymer hydrophilicity with respect to PLA and PLGA. Microsphere preparation was performed by double emulsion solvent evaporation method, and an accurate study of the preparation protocol involved a design of experiment (DoE) screening test in order to optimize gentamicin sulphate loading into microspheres. The results of in vitro gentamicin sulphate release showed prolonged gentamicin release, up to three months, from the microspheres prepared with NaCl addition in the dispersing phase; this behaviour was consistent with the compact microspheres’ structure. Prolonged release of gentamicin sulphate was achieved by loading the drug in PLGA-PEG microspheres, and it was maintained after embedding the biodegradable microspheres into mCS. Since gentamicin sulphate release from mCS happened in 4 h by diffusion through the chitosan-based matrix, the authors concluded that gentamicin release control was mainly performed by PLGA-PEG, while chitosan matrix acted as support for the microspheres. The composite scaffold was able to retain the microspheres for up to 40 days. Indeed, retention capability depended on the amount of microspheres loaded into the scaffold. Microbiological evaluation demonstrated the efficacy of gentamicin-loaded microspheres on *E. coli*. The collected results suggested high potential of the microparticulate drug delivery system to be used for the local antibiotic delivery to bone [116,117].

The authors’ research on the topic also involved natural polymer and polymer combination of natural origins such as eumelanin and polydopamine. Eumelanin is a heteropolymer that spontaneously self-assembles in round-shaped micelles of nanometric size. The eumelanin nanoparticles were synthesized and functionalized by dopamine self-polymerization, obtaining polydopamine-coated eumelanin nanoparticles (FEUNp), aimed to facilitate conjugation through polydopamine functional groups and to improve eumelanin hydrophilicity. The FEUNp, whose size was about 230.04 ± 8.25 nm with negative charge (−36.60 ± 0.45 ξ potential), were loaded with gentamicin sulphate (FEUNp-GS) by an adsorption process, and studied as drug delivery systems to treat osteomyelitis. The microbial effect of FEUNp-GS on *Staphylococcus aureus* and *Escherichia coli* were tested, demonstrating the sustained release of gentamicin from the FEUNp-GS. The advantage of studying the natural polymer eumelanin resides in its scavenger effect able to react with the free radicals, thus potentially reducing gentamicin toxicity. Moreover, polydopamine has bioadhesive properties that can be useful when formulating the FEUNp-GS in gels or mCS to be locally administered [119].

Looking towards the future, literature reports antibiotic-loaded nanoparticles as a promising drug delivery system (DDS) to be combined with scaffold for bone regeneration, because they seem to improve antibiotic efficacy even against bacterial biofilms. According to their sub-micron size, nanoparticles efficiently cross biological barriers, and they can improve drug bioavailability and drug permanence at the infected site. Moreover, nanoparticles protect the drug from degradation and can achieve a gradual drug release pattern with improved antibiotic effect. As an example, Abdelghany and co-workers prepared gentamicin-loaded PLGA nanoparticles and tested them against *Pseudomonas aeruginosa* PA01, both planktonic and biofilm cultures, as long as on a peritoneal murine infection model. The nanoparticles demonstrated significant improvement of antimicrobial effect, above all in the murine model, as indicated by a reduction of inflammatory indicators interleukin-6 and myeloperoxidase [81]. Literature on polymer and metal nanoparticles shows that many questions are still open about the antibacterial mechanism of nanoparticles, also depending on the material used to make them [78,79,83,120,121]. Moreover, an issue related to nanoparticles loaded with antibiotics is the usually low drug content if compared with the high antibiotic dose regimens required [78,79,80,81,82,83]. Nanoparticulate drug delivery systems can be advantageously loaded into polymer scaffold for tissue regeneration. The author’s research is in progress to evaluate the antimicrobial activity of PLGA-PEG nanoparticles loaded with gentamicin sulphate, and further formulation studies into mSC will be conducted involving FEUNp-GS and gentamicin-loaded PLGA-PEG Nps.

## 6. Conclusions

The wide literature on the topic demonstrates that combining scaffold for tissue regeneration with drugs—especially drug delivery systems—is a suitable strategy to improve tissue regeneration. Studies are addressed to find biomaterials—or a combination of biomaterials—with properties suitable to support tissue regeneration, and to obtain drug delivery systems able to modulate drug release. Such scaffold/drug delivery systems are addressed to combine several advantages, namely: (i) local drug delivery with improved bioavailability and reduced adverse effects with respect to systemic drug administration; (ii) sustained drug release; (iii) ability of combining two or more drugs in a single scaffold.

The case of osteomyelitis represents an example where a severe infection can be treated efficiently with locally delivered antibiotic combined to biodegradable polymer scaffold for bone regeneration. In a future perspective, nanoparticles represent an interesting tool to improve antibiotic efficacy, also reducing bacterial resistance.

The topic poses the question of regulatory aspects related to such combination products, whether they should be considered as drug products or medical devices. The issue is beyond the scope of this brief review; however, it needs to be considered by companies wishing to place these products on the market.

## Abbreviations

BBS	Bovine bone substitute granules
rhBMP-2	Bone Morphogenetic Protein 2 recombinant
BCP	Biphasic calcium phosphate
CHS	Chitosan
CMC	Carboxymethyl cellulose
DDS	Drug delivery system
DoE	Design of experiment
ECM	Extra Cellular Matrix
EMA	European Medicines Agency
FDA	Food and Drug Administration
FEUNp	Polydopamine-coated eumelanin nanoparticles
FEUNp-GS	Gentamicin sulphate loaded polydopamine coated eumelanin nanoparticles
GFs	Growth factors
β-GP	β-glycerophosphate
HA	Hyaluronic acid
HAP	Hydroxyapatite
HCA	Hydrocarbonate apatite
ITCS	Injectable thermosetting composite scaffolds
mCS	3D moldable composite scaffold
Nps	Nanoparticles
PANH	Poly (1,6-bis-(*p*-carboxyphenoxy hexane)-*co*-(sebacic anhydride)
PCL	Polycaprolactone
PCL-gHAP	poly(ɛ-caprolactone) grafted to hydroxyapatite (PCL-gHAP)
PGA	Polyglycolic acid
PLA	Polylactide
PLGA	Polylactide-*co*-glycolide
PLGA-PEG	Polylactide-*co*-glycolide-*co*-polyethylenglycol
PMMA	Polymethylmethacrylate
PPF	Poly(propylene fumarate
β-TCP	β-tricalcium phosphate
TE	Tissue engineering
ZIF-8	Zeolitic imidazolate framework-8
